# Distributed compressed sensing to accelerate cine cardiac MRI

**DOI:** 10.1186/1532-429X-17-S1-P391

**Published:** 2015-02-03

**Authors:** Jafar Zamani, Abbas N Moghaddam, Hamidreza S Rad

**Affiliations:** 1 Biomedical Engineering, Amirkabir University of Technology - Tehran Polytechnic, Tehran, Iran (the Islamic Republic of; 2 Medicine, David Geffen School, Los Angeles, CA, USA; 3 Medical Physics and Biomedical Engineering, Tehran University of Medical Sciences (TUMS), Tenran, Iran (the Islamic Republic of; 4 Quantitative MR Imaging and Spectroscopy Group, Research Center for Molecular and Cellular Imaging, Tehran University of Medical Sciences (TUMS), Tenran, Iran (the Islamic Republic of

## Background

Compressed sensing (CS) is an efficient tool that accelerates the data acquisition in MRI through the significant reduction of required measurements for image reconstruction. In recent years, there have been significant interests in the use of Compressed Sensing (CS) in Dynamic applications [1]. Since Cine cardiac images, as a dynamic data, has both spatial and temporal sparsity, it is a good candidate for CS with high acceleration factor [2].

## Methods

**Figure 1:**
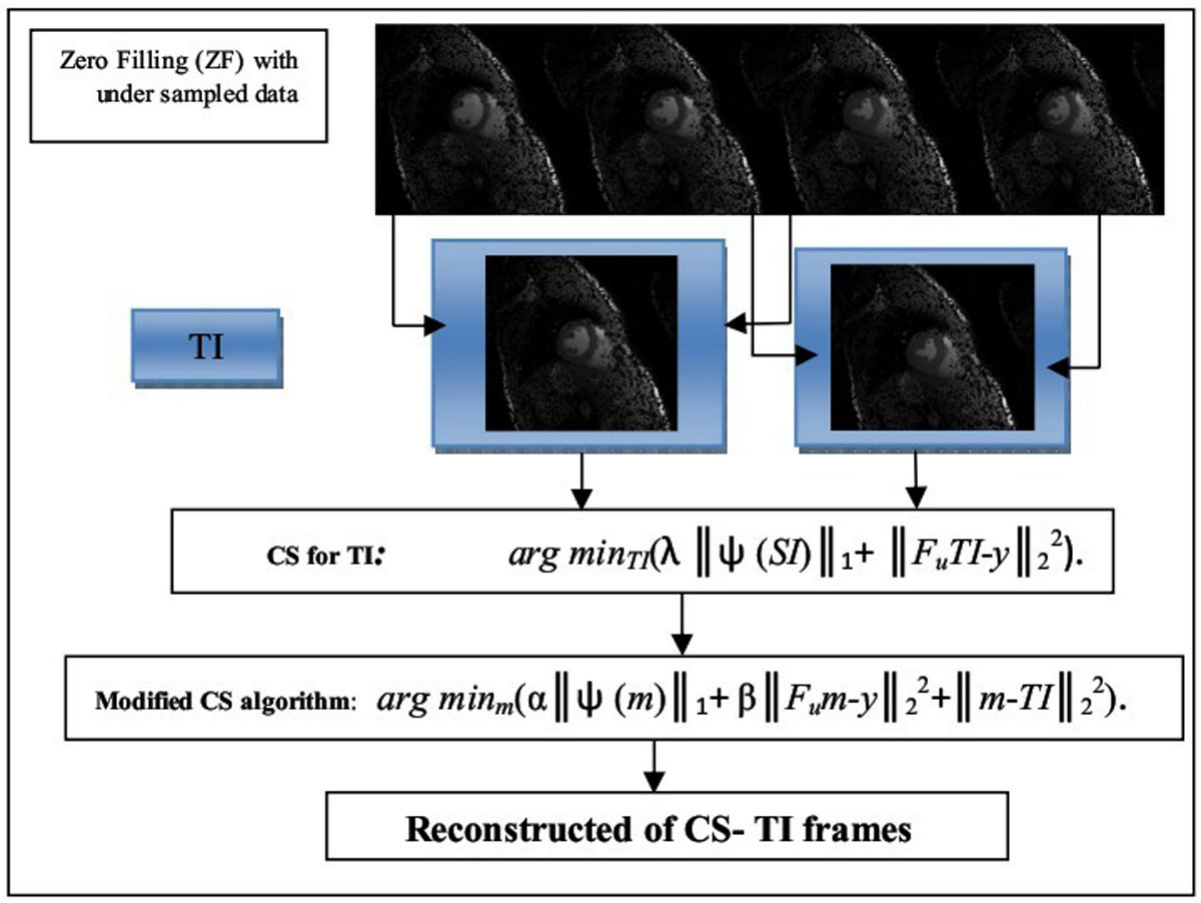
The flowchart of proposed method.

In this study we obtain Temporal Estimation (TE) from Temporal Information (TI) using forward-backward motion estimation (F-B ME) to highly accelerate dynamic MRI acquisition.We implement CS on TE in the first step and then use these reconstructed frames with under-sampling K-space data in aModified CS (MCS) scheme. Figure 1 illustrates our proposed method in details. In MCS we add another fidelity term to conventional CS to penalize the error between TE and the reconstructed frame at each iteration. The gradient projection for sparse reconstruction (GPSR) was exploited to achieve faster solutions and fair quality on frames reconstruction. The vicinity of center at the Fourier space domain was chosen completely and to satisfy incoherent condition of under-sampling in CS, other parts of k-space were sampled using a random Gaussian probability function, with Discrete Wavelet Transform (DWT) sparsifying basis. Total Sampling rate was set to 0.2. Experiments were performed on a data set acquired and provided by Cagdas Bilen et al. [3]. Data were acquired using a 128×128 matrix (FOV = 320 × 320 mm) and 23frames covering the cardiac cycle.

## Results

**Figure 2:**
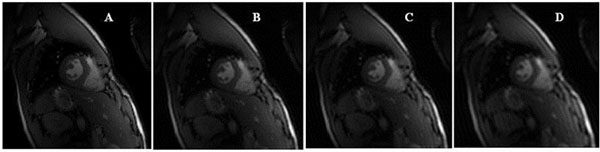
A)Original image from full K-space data, B) proposed method with 20% randomunder-sapmling K-space data, C)CS_TI with proposed method data, D) CS with proposed method data.

The results of the proposed algorithm were compared to those of conventional CS, CS -TI [2] and original image with full k-space data. Figure 2 illustrates one frame from the original set along with the corresponding CS, CS-TI and proposed method. We obtained SNR values of 27.74, 25.13 and 24.32 for proposed method, CS-TI and CS respectively. Finally the SSIM values were 97.24, 91.17 and 87.36 for those three methods respectively which shows the proposed method outperforms CS_TI and CS respectively in the quality measures.

## Conclusions

We proposed a new method to highly accelerate Cine cardiac images using spatial and temporal sparsity based on CS theory. The proposed method high efficiency under-sampling rate and fidelity in CS theory. The results show the efficiency and accuracy of the proposed methods compared to other conventional CS methods. Concluding that proposed method outperforms other methods in both SNR and SSIM with 10% and 8%, respectively.
